# Molecular and metabolic alterations of 2,3-dihydroquinazolin-4(1*H*)-one derivatives in prostate cancer cell lines

**DOI:** 10.1038/s41598-022-26148-4

**Published:** 2022-12-14

**Authors:** Lina A. Dahabiyeh, Wafa Hourani, Wesam Darwish, Farah Hudaib, Bashaer Abu-Irmaileh, Pran Kishore Deb, Katharigatta N. Venugopala, Viresh Mohanlall, Rana Abu-Dahab, Mohammad H. Semreen, Yasser Bustanji

**Affiliations:** 1 Department of Pharmaceutical Sciences, School of Pharmacy, The University of Jordan, Queen Rania St, Amman, 11942 Jordan; 2 Department of Pharmaceutical Sciences, Faculty of Pharmacy, Philadelphia University, Amman, 19392 Jordan; 3 Depatment of Pharmaceutical Chemistry, School of Pharmaceutical Sciences, the Hashemite University, Zarqa, 13133 Jordan; 4 Hamdi Mango Center for Scientific Research, The University of Jordan, Amman, 11942 Jordan; 5 Department of Pharmaceutical Sciences, College of Clinical Pharmacy, King Faisal University, Al-Ahsa, 31982 Saudi Arabia; 6 Department of Biotechnology and Food Science, Faculty of Applied Sciences, Durban University of Technology, Durban, 4000 South Africa; 7 Department of Biopharmaceutics and Clinical Pharmacy, School of Pharmacy, The University of Jordan, Amman, 11942 Jordan; 8 Department of Medicinal Chemistry, College of Pharmacy, University of Sharjah, Sharjah, 27272 UAE; 9 Department of Basic Medical Sciences, College of Medicine, University of Sharjah, Sharjah, 27272 UAE

**Keywords:** Cancer, Molecular biology, Systems biology, Biomarkers, Oncology, Chemistry

## Abstract

Prostate cancer (PC) is the second most common tumor in males worldwide. The lack of effective medication and the development of multidrug resistance towards current chemotherapeutic agents urge the need to discover novel compounds and therapeutic targets for PC. Herein, seven synthesized 2,3-dihydroquinazolin-4(1*H*)-one analogues were evaluated for their anticancer activity against PC3 and DU145 cancer cell lines using MTT, scratch-wound healing, adhesion and invasion assays. Besides, a liquid chromatography mass spectrometry (LC–MS)-based metabolomics approach was followed to identify the biochemical pathways altered in DU145 cancer cells upon exposure to dihydroquinazolin derivatives. The seven compounds showed sufficient cytotoxicity and significantly suppressed DU145 and PC3 migration after 48 and 72 h. C2 and C5 had the most potent effect with IC_50_ < 15 µM and significantly inhibited PC cell adhesion and invasion. Metabolomics revealed that C5 disturbed the level of metabolites involved in essential processes for cancer cell proliferation, progression and growth including energy production, redox homeostasis, amino acids and polyamine metabolisms and choline phospholipid metabolism. The data presented herein highlighted the importance of these compounds as potential anticancer agents particularly C5, and pointed to the promising role of metabolomics as a new analytical approach to investigate the antiproliferative activity of synthesized compounds and identify new therapeutic targets.

## Introduction

Prostate cancer (PC) is the second most common tumor in males globally, following lung cancer^1,2^. In 2018, PC accounted for 3.8% of all deaths caused by cancer in men^2^. The global incidence and death rate of prostate cancer increase with age; with the average age at diagnosis being 66 years^1^. Despite the advances in understanding the etiology and the pathogenesis of PC, and the recognized high risk of men being diagnosed with the illness over their lifetime, an effective medication that can be safely delivered to favorably influence men's lives continues to be lacking^3^. There are different approaches to treat PC including radiotherapy, cryotherapy, hormone therapy, chemotherapy, immunotherapy, targeted therapy with surgery being the most common treatment for PC^4,5^. Although current treatments can improve the survival rate and the outcomes in men with metastatic prostate cancer, they all come with side effects such as erectile dysfunction and urinary incontinence which affects the patient’s quality of life^4,5^. Additionally, the development of multidrug resistance towards current hormonal therapy and chemotherapeutic agents urges the need to discover novel compounds and targets for prostate cancer therapy^6^.

Quinazoline derivatives are promising heterocyclic compounds and a fundamental structural-nucleus in a variety of therapeutic molecules and physiologically active substances^7^. They are of great interest due to their broad spectrum of pharmacological activities and favorable side effects profile^8^. Quinazoline derivatives have been reported as anticancer, antioxidant, antiviral, anticonvulsant, larvicidal, anti-inflammatory, and antitubercular compounds^8–12^. The Food and Drug Administration (FDA) has approved several quinazoline derivatives for clinical use as anticancer drugs. This includes gefitinib, for treating locally advanced or metastatic non-small-cell lung cancer (NSCLC), erlotinib, for NSCL and pancreatic cancer treatment and lapatinib, for breast cancer treatment^10,13^. Moreover, in vitro and in vivo studies have proven the anticancer activity of quinazoline derivatives against different cancer cells, including human prostate cells; DU145, LNCaP, and PC-3^14^.

The development of novel quinazoline derivatives as promising anticancer drug candidates is receiving increasing interest worldwide. However, most of the current research focuses on evaluating the antiproliferative activity of these compounds using classical biochemical assays^15^. Cancer cells are well-known to have changes in some metabolic pathways^16^ . Therefore, it is of high importance to evaluate the effect of these compounds on metabolites levels and biochemical pathways in cancer cells to provide an insight into their underlying molecular mechanisms. Metabolomics is an advanced analytical approach for qualitative and quantitative analysis of small molecules (referred to as metabolites) in a specific biological sample^17,18^. It is an effective strategy to identify biomarkers associated with pathological conditions and metabolic signatures linked to drug exposure^19–22^. In PC, metabolomics has been used to differentiate between malignant PC and benign tissue^23^, classify different PC cell lines^24^, and identify biomarkers related to disease progression and treatment response^25^.

In this work, the cytotoxicity of seven synthesized 2,3-dihydroquinazolin-4(1*H*)-one analogues was evaluated, using MTT assay, against two human androgen-independent PC cell lines namely; PC3 and DU145. In vitro scratch-wound healing assay was performed to investigate the effect of the compounds on the migration ability of the DU145 and PC3 cell lines. The most promising compounds were further evaluated using adhesion and invasion assays, and a liquid chromatography mass spectrometry (LC–MS)-based metabolomics approach was followed to identify the biochemical pathways altered in DU145 cancer cells upon exposure to dihydroquinazolin derivatives and detect novel therapeutic targets.

## Materials and methods

### Synthesis of 2,3-dihydroquinazolin-4(1*H*)-one analogues

The seven 2,3-dihydroquinazolin-4(1*H*)-one analogues (C1-C7) were synthesized according to our previous protocol^11^. Stock solutions of the compounds were prepared in culture grade dimethyl sulfoxide (DMSO) (Santa Cruz Biotechnology, Dallas, TX, USA) and diluted using cell culture media. The final concentration of DMSO in the tested concentrations did not exceed 0.5%.

### Cell lines

Human PC cell lines; PC3 and DU145 were obtained from the American Type Culture Collection (ATCC) (Manassas, VA, USA). PC3 and DU145 cell lines were cultured in RPMI-1640 high glucose media with L-glutamine (Euroclone, Italy), supplemented with 10% fetal bovine serum (FBS) (Ebsdorfergrund, Germany) and 1% (*v/v*) penicillin–streptomycin (Euroclone, Italy). Cell cultures were incubated at 37 °C, 5% CO_2_ with 95% humidity. Both cell lines were passaged twice a week at 70–90% confluence.

### Cell viability assay

The cytotoxic effects of the seven dihydroquinazolin-4(1*H*)-one derivatives on the two PC cell lines were measured using the MTT (3-[4,5-Dimethylthiazol-2-yl]-diphenyltetrazolium Bromide) colorimetric assay kit (Promega, Madison, WI, USA). PC3 and DU145 cells were seeded in a 96-well plate at seeding densities of 10 × 10^3^ and 6 × 10^3^ cells/well, respectively. After overnight incubation at 37 °C and 5% CO_2_, cells were treated with various concentrations of the seven compounds to determine their half maximum inhibitory concentrations (IC_50_) values, and plates were incubated at 37 °C for 72 h. After incubation, the culture medium was removed and 100 μL RPMI (containing 5% FBS and 1% penicillin–streptomycin) and 15 μL dye solution were added to each well and the plate was incubated at 37 °C for 3 h. At the end of the incubation, 100 μL solubilization solution was added, and after complete solubilization of the MTT dye, the absorbance was measured at 570 nm using Multiskan Go Microplate Reader (ThermoFisher Scientific, Hampshire, UK). Cell viability was calculated according to the formula below:

$$\mathrm{Cell\, viability }(\mathrm{\%})=\frac{\mathrm{Absorbance \,of\, treated\, cells}-\mathrm{Blank\, Absorbance}}{\mathrm{Absorbance\, of\, untreated\, cells}-\mathrm{Blank\, Absorbance}}\times 100$$

### Wound healing scratch assay

Human PC cell lines, PC3 and DU145, were seeded at a density of 16 × 10^4^ cells/well in a 12-well plate using 4 mL of supplemented RPMI, then the cells were incubated for 24 h at 37 °C, 5% CO_2_ until confluency 80–90%. A scratch was induced into the cells using the tip of the micropipette, and the wells were treated with sub-IC_50_ concentrations of the seven compounds (the concentrations that showed 80% survival rates in comparison to control on the cellular viability assay (IC_20_) were used in this experiment). Scratched untreated cells served as a control. Pictures of scratched cells were captured at different time points (0, 24, 48 and 72) hr post treatment using a light microscope (BOECO, Germany) coupled with a 5.0 Mega Cmos camera at a magnification of 40X. Three independent experiments were performed. The distance between the two sides of the scratch was measured and analyzed using Image J-image analysis software (NIH, Bethesda, MD, USA), and the percentage of migration inhibition was calculated using the equation below:

$$\mathrm{Migration\, Inhibition }(\mathrm{\%})=\frac{\mathrm{Distance\, between\, wound\, edges\, at\,}24, 48\mathrm\,{or}\,72\mathrm\,{hr}}{\mathrm{Distance\, between\, wound\, edges\, at\, zero\, time}}\times 100$$

### Adhesion assay

Sub-IC_50_ concentrations of the selected compounds (IC_20_; C2 and C5) were used for the adhesion and invasion assays. Prior to the assay, cells were cultured in RPMI containing 10% FBS to 80% confluency, then cells were washed with PBS and incubated for 24 h with serum free media containing IC_20_ concentrations of the selected compounds. A 96-well plate was coated with 50 µL of fibronectin (SigmaAldrich, St.Louis, MO, USA) and incubated at 37 °C overnight. The following day; the plate was blocked with 50 µL of 0.2% bovine serum albumin (Sigma, USA) for 2 h at room temperature. The cells were harvested with trypsin/EDTA and resuspended in RPMI at approximately 125,000 cells/mL, and 100 µL of the cell suspension was added to each well pre-coated with fibronectin. The experiment was repeated in triplicates for both the treatment and control. For the control, the same concentration of DMSO in the final dilutions of the compound was used. The plate was then incubated for 30 min at 37 °C. After the incubation period, the wells were gently aspirated and washed three times with PBS, then MTT assay was performed to quantify the cells.

### Invasion assay

Prior to the assay, PC3 and DU145 were grown to 80% confluency and starved in serum free media for 24 h. Cell invasion assay was performed using Trevigen Cultrex® 96 insert cell invasion/ migration chamber kit from Sigma Aldrich as per manufacturer protocol. Briefly, the upper chambers of the 96-well plate were coated with 50 µL 0.6 × BME solution and the plate was incubated at 37 °C overnight. Next day, cells were harvested with trypsin/EDTA and centrifuged for 10 min at 1400 rpm. The supernatant was removed and cells were washed with PBS and resuspended in serum-free media to an approximately 1 × 10^6^ cells/mL. The plate was carefully aspirated, and 50 µL of the cell suspension was added to each well (upper chamber). To the bottom chamber of each well, 150 µL of media supplemented with 10% FBS was added. Finally, the two compounds (at concentration IC_20_) were added to the cell-containing wells. After 48 h incubation, the upper chambers were carefully aspirated, washed with cell wash buffer and 150 µL of Calcein-AM solution was added to each well and incubated for one hour. Fluorescence was measured at 485 nm excitation, 520 nm emission using multi-mode reader (BioTek, Winooski, VT, USA).

### Flow cytometry of DU145 cancer cell line after treatment with 2,3-dihydroquinazolin derivatives

The mode of cell death in DU145 cells treated with selected compounds (C2, C5 and C6) was determined by Annexin V/PI stain using flow cytometry. DU145 cells were seeded in T25 flasks at a density of 5 × 10^5^ cells/flask. Once confluent, each flask was treated with the IC_50_ or double the IC_50_ concentrations of the three compounds, or left untreated to serve as a control. After 72 h incubation at 37 °C and 5% CO_2_, cells were trypsinized by trypsin EDTA (0.25%) and the collected cells were centrifuged for 5 min at 1500 rpm. The formed pellets were washed with PBS twice with a centrifugation step after every wash. After that, the Annexin V/PI apoptosis kit (Abcam, UK) was used to stain the cell pellets following the manufacturer’s instructions. Samples were analyzed using BD FACSCanto II flow cytometer (BD Biosciences, San Jose, CA, USA) and BD FACSDiva software (BD Biosciences, USA).

### Statistical analysis

Data analyses for cell viability assay, wound healing and flow cytometry were performed using GraphPad Prism software version 8 (GraphPad, San Diego, CA, USA). A non-linear regression analysis was applied in the calculation of IC_50_ values. For group comparison, One-way ANOVA analysis using post hoc Tukey’s test was conducted. Student’s independent t-test was used to compare the cell viability between the two cell lines. A *P-*value (< 0.05) was considered significant, where ^*^P < 0.05, ^**^P < 0.01, ^***^P < 0.001, and ^****^P < 0.0001. All experiments were performed in replicates of independent experiments, and results were presented as the mean ± standard deviation (SD).

### Metaboliets Profiling of DU145 cell line treated with C5 and C6 using LC-HRMS/MS

#### Sample preparation and metabolites extraction

Global metabolomic profiling was performed to investigate the molecular mechanisms by which 2,3-dihydroquinazolin derivatives might exert their cytotoxic effect against DU145. DU145 cells were cultured in (100 mm × 20 mm) cell culture Petri dishes (Corning®, NY, USA) at a density of 130 × 10^3^ cell/cm^2^, and incubated in 7 mL fully supplemented RPMI medium for 72 h. After reaching 85% confluence, cells were treated with sub-inhibitory concentrations of compounds C5 and C6 (5 μM and 55 μM, respectively), or left without treatment to serve as controls (n = 10 in each group). Cells were incubated for 4 h at 37 °C, then the treatment was replaced by a complete RPMI medium, and cells were incubated for 20 h. At the end of the 20 h, the culture medium was removed, and cells were washed with a pre-warmed (37 °C) phosphate buffer saline (PBS) (Euroclone, Italy). The cell metabolism was quenched with 0.5 mL pre-cooled (−48 °C) LC–MS grade methanol (ChemLab, Belgium), and extracellular metabolites were extracted as described previously^20^.

#### Metabolite profiling using liquid chromatography-mass spectrometry (LC–MS/MS)

Dried samples were resuspended in 200 µL 0.1% formic acid (FA) (Fisher Chemical, Belgium) in deionized Water LC–MS CHROMASOLV (Honeywell, Germany), vortexed for 2 min, and filtered using a hydrophilic nylon syringe filter of 0.45 µm pore size to be analyzed by liquid chromatography-mass spectrometry (LC–MS/MS). Quality control (QC) sample was prepared by pooling the same volume (10 µL) of each sample, and all samples were placed in the autosampler. Extracted metabolites were separated using Elute UHPLC (Bruker Daltonik GmbH, Bremen, Germany) and then profiled using quadrupole time-of-flight mass spectrometer (Q-TOF MS) (Bruker Daltonik GmbH, Bremen, Germany). Windows 10 Enterprise 2016 LTSB was used as the computer operating system. The data management software was Bruker Compass HyStar 5.0 SR1 Patch1 (5.0.37.1), Compass 4.1 for otofSeries, otof Control Version 6.2.

Metabolites were eluted in Intensity Solo C18 column (2.1 × 100 mm, 1.8 µm) (Bruker Daltonik), using solvent A (0.1% FA in LC–MS grade water) and solvent B (0.1% FA in LC–MS grade acetonitrile) with the following gradient elution mode: 0–2 min, 1% B; 2–17 min, 1–99% B; 17–20 min, 99% B; 20–20.1 min, 99–1% B; 20.1–30 min, 1% B. The flow rate was 0.25 mL/min. The autosampler and column oven temperatures were kept at 4 °C and 35 °C, respectively. A total volume of 10 µl was injected into the Q-TOF MS. For ion detection, the drying gas flow rate was 10.0 L/min, the End Plate offset was set at 500 V, the nebulizer pressure of 2.2 bar, the electrospray ionization (ESI) capillary voltage and the drying temperature were 4500 V and 220 °C, respectively. Mass spectra were obtained in a data-dependent manner automatically changing among MS and MS/MS scans within the range of 20–1300 m/z. The collision energy stepping was between 100 and 250% of set at 20 eV. The acquisition involved two segments; auto MS scan, which ranged from 0 to 0.3 min for the calibrant sodium formate, and auto MS/MS scan with CID acquisition, which included fragmentation and ranged from 0.3 to 30 min. The acquisition in both segments was performed using the positive mode at 12 Hz and the width of the precursor ion was ± 0.5, the number of precursors was 3, the cycle time was 0.5 s., and the threshold was 400 cts. Active exclusion was excluded after 3 spectra and released after 0.2 min. Mass calibration was done prior to analysis according to the manufacturer’s recommendations using external mass calibration (10 mM sodium formate calibrant solution). A test mixture of TRX-2101/RT-28-calibrants for Bruker T-ReX LC-QTOF solution from Nova Medical Testing Inc. was used to check the performance of LC separation and perform multipoint retention time calibration. The TRX-3112-R/MS Certified Human serum for Bruker T-ReX LC-QTOF solution; a product prepared from pooled human blood and provided by Nova Medical Testing Inc. was used to check the performance of sample preparation protocols as well as LC–MS instruments, as recommended by the manufacturer. The analysis was performed using a randomized sequence order with five injections of solvent A sample at the beginning of the sequence for apparatus equilibration, followed by five injections of the pooled QC sample. Additionally, one QC injection was performed every (9–10 samples) to evaluate the reproducibility of the analysis.

#### Data processing and metabolites identification

Data processing was performed using MetaboScape® 4.0 software (Bruker Daltonics). The data were processed for untargeted peak alignment, peak-picking, and annotation of related peaks. For bucketing in T-ReX 2D/3D workflow, the parameters set for molecular feature detection were as follows: minimum intensity threshold equal to 1000 counts along with minimum peak length of 7 spectra for peak detection, using peak area for feature quantitation. The mass recalibration was done within a retention time range between 0 and 0.3 min. The detected MS/MS spectra were assigned to the bucket table and included retention time, measured *m/z*, detected fragments and molecular weight. On the other hand, the MS/MS import method was set to be done by average spectrum out of all MS/MS spectra. The parameters for data bucketing were assigned as follows: retention time range started at 0.3 min and ended at 25 min, while mass range started at 50 m/z and ended at 1300 m/z.

The annotation was performed using the Human Metabolome Database (HMDB-4.0) spectral library. The Annotation Quality (AQ) score indicator was set at *m/z* 2.0–5.0 mDa, retention time 0.1–0.4 min, mSigma 10–20, MS/MS score 900–800, CCS 2.0–5.0%.

#### Statistical analysis (uni- and multivariate analysis)

Processed data (mass ions with their normalized abundances) were exported for multivariate analysis using Simca P + 14 (Sartorius Stedim Data Analytics AB, Umea, Sweden). The datasets were centered around the mean and pareto scaled. For assessing the analytical performance of LC–MS/MS run, the unsupervised principal component analysis (PCA) with quality control (QC) samples was used. Modelling the differences among the studied groups was achieved by partial least square-discriminative analysis (PLS-DA) and orthogonoal PLS-DA (OPLS-DA). The robustness of the created models was evaluated by the model fitness (R^2^X) for PCA, and (R^2^Y) for PLS-DA and OPLS-DA, as well as monitoring the predictive ability (Q^2^) values. An acceptable model is considered when a model yields large R^2^X and R^2^Y values; (values close to 1) and Q^2^ values > 0.5. OPLS-DA models were further validated using a permutation test (100 permutations). Variable importance in the projection (VIP) more than 1 was employed to select the significant mass ions accountable for the class separation between the compared groups in the generated OPLS-DA scores plot^26^.

Univariate analysis was performed by uploading the processed raw data to MetaboAnalyst version 5.0 (McGill University, Montreal, QC, Canada)^27,28^. Datasets were normalized to sample total median and pareto-scaled. An Independent t-test was used to identify significantly altered mass ions/metabolites. A false discovery rate (FDR) less than (0.05) is defined as significant. Volcano plots were visualized applying FDR values less than 0.05 with fold changes (FC) cuttoff of 1.5. Significantly altered metabolites determined based on their multivariate analysis (VIP > 1.0) and univariate analysis (FDR < 0.05) were subjected to biochemical pathways analysis.

## Results

### Synthesis and characterization of 2,3-dihydroquinazolin-4(1*H*)-one analogues

**Scheme 1:**
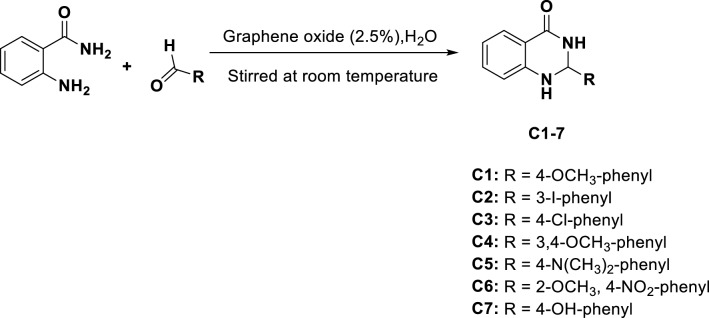
Synthetic scheme for the construction of 2-(substituted phenyl)-2,3-dihydroquinazolin-4(1*H*)-ones (**C1–C7**).

Seven 2,3-dihydroquinazolin-4(1*H*)-one derivatives (C1-C7) were synthesized as depicted in Scheme 1. The yield of the title compounds was in the range of 82–95%, and the purity was > 99%, as determined by the HPLC method. Characterization of the compounds, including FT-IR, ^1^H, and ^13^C-NMR spectra is fully described in our previouse work^11^. The same characterized compounds were used in the current work. The molecular structures of the seven dihydroquinazolin derivatives are shown in Table 1.

**Table 1:** Cytotoxic effect of the seven substituted 2,3-dihydroquinazolin-4(1H)-ones analogues (C1-C7) against PC3 and DU145 prostate cancer cell lines.

Cmp	Scientific name	Compound structure	Cytotoxicity% at 100 µM*		IC_50_*	
			PC3	DU145	PC3	DU145
C1	2-(4-Methoxyphenyl)-2,3-dihydroquinazolin-4(1*H*)-one		47.4 ± 1.2	60.0 ± 4.6	82.9 ± 4.6	67.6 ± 2.8
C2	2-(3-Iodophenyl)-2,3-dihydroquinazolin-4(1*H*)-one		63.7 ± 2.1	70.9 ± 2.8	14.8 ± 5.3	8.7 ± 1.7
C3	2-(4-Chlorophenyl)-2,3-dihydroquinazolin-4(1*H*)-one		60.4 ± 3.3	69.1 ± 4.1	36.3 ± 3.2	19.3 ± 3.4
C4	2-(3,4-Dimethoxyphenyl)-2,3-dihydroquinazolin-4(1*H*)-one		56.3 ± 3.2	52.7 ± 5.7	77.6 ± 4.3	70.8 ± 3.5
C5	2-(4-(Dimethylamino)phenyl)-2,3-dihydroquinazolin-4(1*H*)-one		73.2 ± 4.1	78.3 ± 4.0	1.1 ± 3.5	2.1 ± 2.3
C6	2-(2-Methoxy-4-nitrophenyl)-2,3-dihydroquinazolin-4(1*H*)-one		45.2 ± 5.1	73.6 ± 3.4	81.3 ± 5.2	50.1 ± 3.2
C7	2-(3-Hydroxyphenyl)-2,3-dihydroquinazolin-4(1*H*)-one		70.7 ± 0.9	53.2 ± 2.2	31.6 ± 3.6	9.8 ± 3.4

*Data are presented as mean ± SD of three replicates of three independent experiments.

### Antiproliferative activity of dihydroquinazolin derivatives

**Figure 1:**
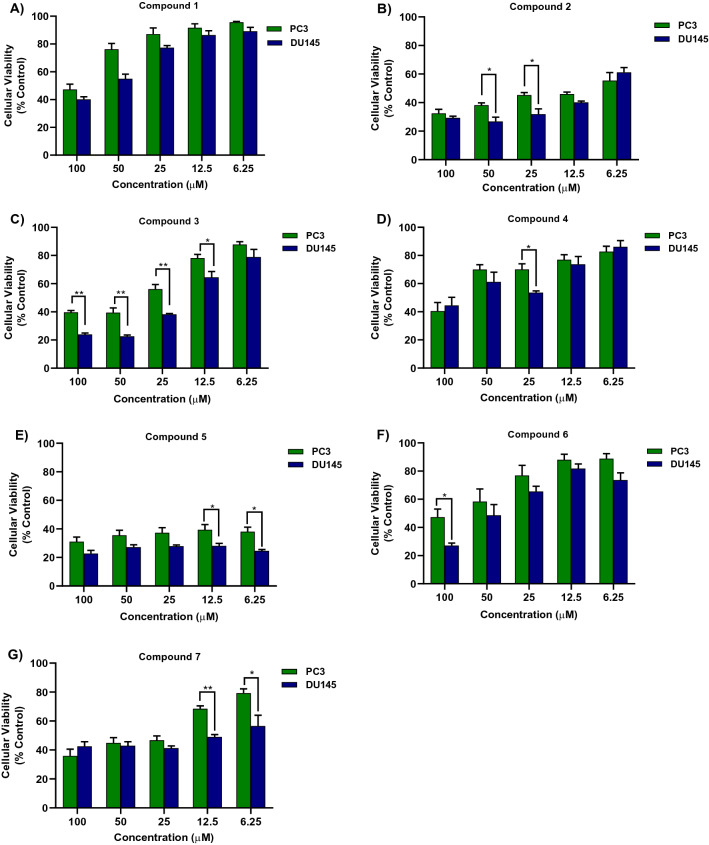
The antiproliferative activities of the seven 2,3-dihydroquinazolin-4(1*H*)-one analogues (C1–C7) against PC3 (green) and DU145 (blue) cell lines. (**A**–**G**) refer to C1, C2, C3, C4, C5, C6 and C7, respectively. Significance between two cell line is expressed as ^*^P-value ≤ 0.05, ^**^P-value ≤ 0.01 (Student’s independent t-test).

Figure 1 shows the antiproliferative effect of different concentrations of dihydroquinazolin derivatives against PC3 and DU145 cell lines. After 72 h incubation, the seven dihydroquinazolins demonstrated a concentration-dependent cytotoxicity in both cell lines. Some compounds showed significant differences between the two cell lines as noted in Fig. 1. C3 showed the most significant difference between the two cell lines with a higher cytotoxic effect against DU145 compared to PC3. Notably, C5 was associated with the highest reduction in cell viability in both PC3 and DU145 cells with cytotoxicity at 6.25 µM of nearly 60% and 72%, respectively.

**Figure 2:**
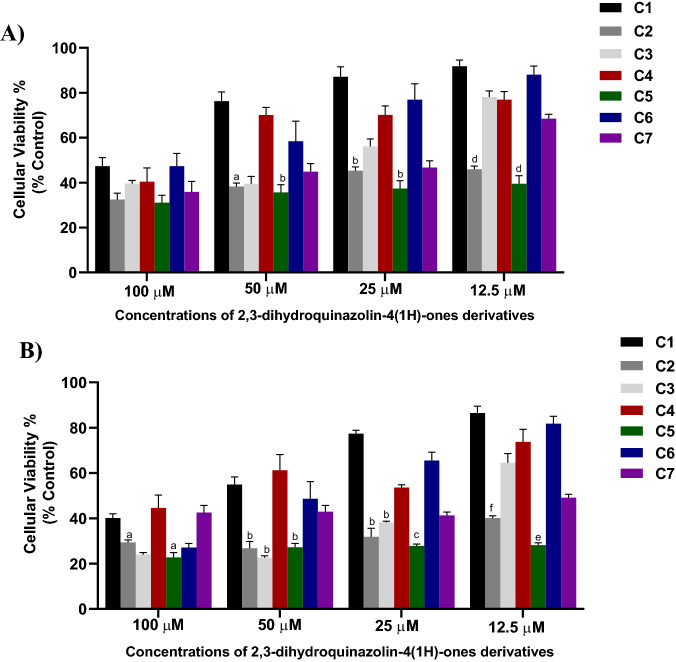
The antiproliferative activities of the seven 2,3-dihydroquinazolin-4(1*H*)-one analogues (C1–C7) against PC3 (**A**) and DU145 (**B**). ^a^Significant difference in comparison to C1 and C4. ^b^Significant difference in comparison to C1, C4, C6. ^c^Significant difference in comparison to C1, C4, C6 and C7. ^d^Significant difference in comparison C1, C3, C4, C6 and C7. ^e^significant difference in comparison to all compounds except C2. ^f^significant difference in comparison to C1, C3, C4 and C6. One-way ANOVA analysis using Tukey’s post hoc test was used to indicate significance (*P*-value ≤ 0.05).

Assessing the effect of the seven dihydroquinazolins against PC3 revealed that all compounds had comparable cytotoxic effects at 100 µM (Fig. 2). However, at lower concentrations (50, 25 and 12.5 µM), C5 and C2 exhibited the highest cytotoxic effect while C1 and C6 showed the least cytotoxic effect compared to the remaining compounds (Fig. 2A). In DU145, C5 had the most pronounced and significant antiproliferative effect compared to the other compounds at all tested concentrations (Fig. 2B). Similar to results on PC3, C1 had the least cytotoxic effect against DU145 (Fig. 2).

The half maximum inhibitory concentrations (IC_50_) of the seven dihydroquinazolin derivatives were comparable against the two cell lines (Table 1 and Fig.S1 in supplementary) with the exception of C7 that showed lower IC_50_ against DU145 (9.8 ± 3.4 µM) compared to PC3 (31.6 ± 3.6 µM). Most derivatives had promising IC_50_ values with C5 and C2 demonstrating IC_50_ in the low micromolar range (< 15 µM).

### Suppression of migration, adhesion and invasion of PC3 and DU145 cell lines

**Figure 3:**
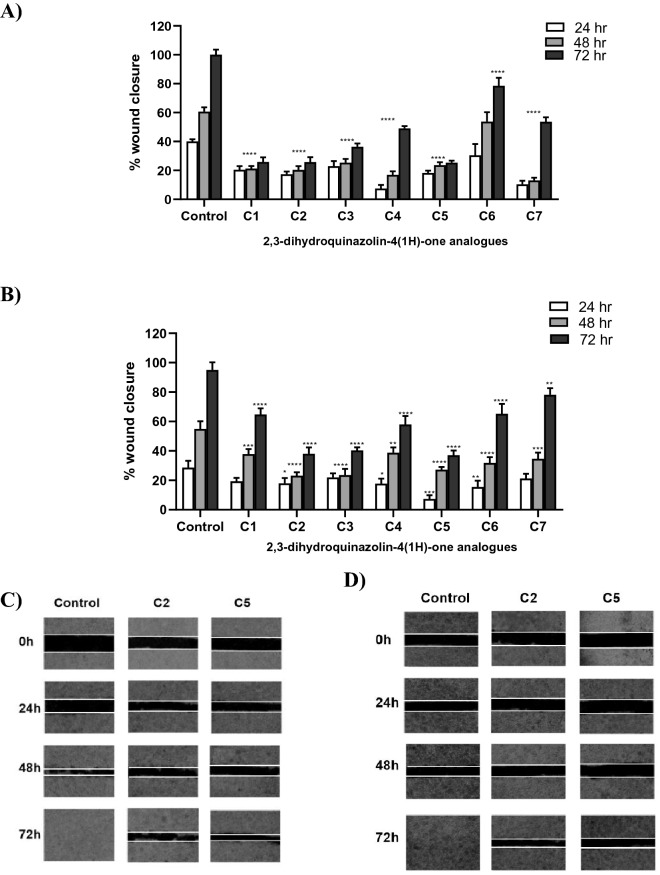
Effect of the seven 2,3-dihydroquinazolin-4(1*H*)-one analogues (C1–C7) on the migration of PC3 and DU145 prostate cancer cell line. Wound healing assay presented as % of wound closure after 24 h, 48 h and 72 h treatment compared to control untreated cells in (**A**) PC3 and (**B**) DU145 cell lines. Effect of C2 and C5 on wound in PC3 (**C**) and DU145 (**D**) cell lines well at time 0 h, 24 h 48 h and 72 h. Data in A and B are shown as mean ± SD of triplicates compared with the untreated control. One-way ANOVA analysis using Tukey’s post hoc test was used to indicate significance. ^*^ *P*-value ≤ 0.05, ^**^P-value ≤ 0.01, ^***^P-value ≤ 0.001, ^****^P-value ≤ 0.0001 compared to control.

Cell migration and invasion are central processes in cancer cell metastasis. Initially, the seven compounds were tested (at sub IC_50_ concentrations) to determine their ability to suppress the migration of PC3 and DU145 cells using wound-healing assay (Fig. 3). In PC3 cancer cells, all compounds significantly suppressed wound closure compared to control at the three-time points with the exception of C6, which only showed a significant difference compared to control after 72 h of treatment (Fig. 3A). In DU145, after 24 h incubation only C2, C4, C5 and C6 displayed a significant reduction in cell migration, while after 48 and 72 h all compounds significantly suppressed DU145 migration (Fig. 3B). In both cell lines, complete closure of the wound in control cells was evident after 72 h, as shown in Fig. 3C and D.

Among the seven compounds, C6 showed the least effect but still could suppress PC3 and DU145 cell migration of nearly 22% and 35% at 72 h. On the other hand, C2 and C5 showed the highest effect in the two cell lines and demonstrated nearly 75% and 62% inhibition of wound closure in PC3 and DU145 cells, respectively, after 72 h treatment (Fig. 3). Therefore, C2 and C5 were further assessed for their potential effects on adhesion and invasion of PC3 and DU145 cells.

**Figure 4:**
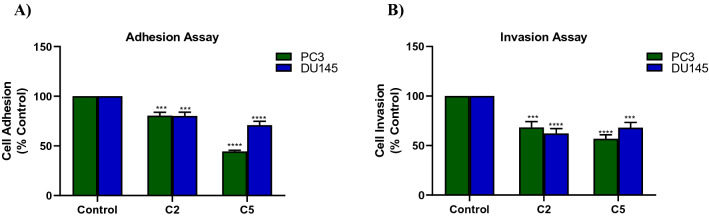
Effects of compounds C2 and C5 on PC3 (Green) and DU145 (Blue) cells adhesion (**A**) and invasion (**B**). Data in A and B are shown as mean ± SD of triplicates compared with the untreated control. One-way ANOVA analysis using Tukey’s post hoc test was used to indicate significance. ^***^P-value ≤ 0.001, ^****^P-value ≤ 0.0001 compared to control.

Compared with untreated cells, both C2 and C5 significantly decreased the number of adherent cells with C5 having a more significant decrease than C2 particularly for PC3 cell line (Fig. 4A). Results of invasion assay showed that treatment with C2 and C5 significantly attenuated PC3 and DU145 cell invasion (Fig. 4B). The two treatments decreased PC cell invasion approximately 32–44% when tested at IC_20_ concentrations.

### Flow cytometry

**Figure 5:**
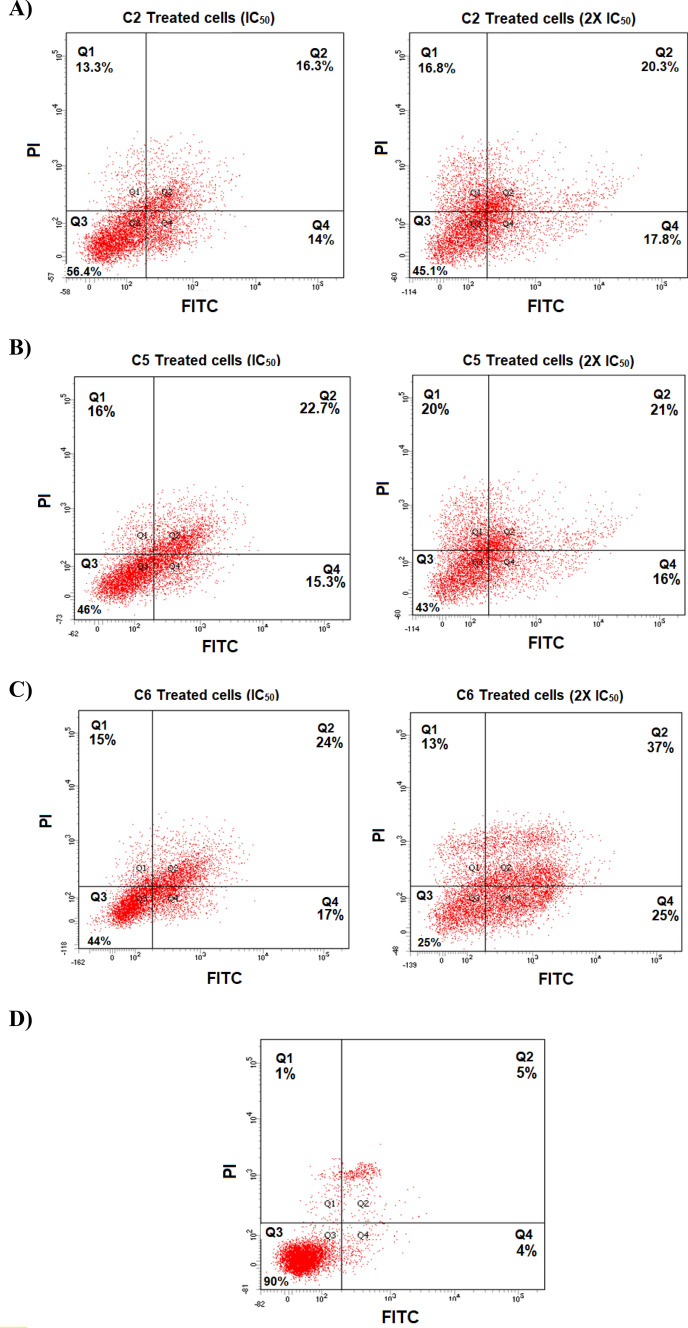
Flow cytometry assay of DU145 cellular death modality. Dot plots of DU145 cells treated with IC_50_ and 2X IC_50_ of C2 (**A**), C5 (**B**), C6 (**C**) or untreated control (**D**). Q1 indicates necrotic cells, Q2 late apoptotic cells, Q3 viable cells and Q4 early apoptotic cells.

The cellular death modality of DU145 cell lines upon exposure to three dihydroquinazolin derivatives (C2, C5 and C6) was studied using flow cytometry. C2 and C5 represented the most active compounds while C6 was among the compounds that showed the least antiproliferative and migration effects. Cells were treated with IC_50_ or 2X IC_50_ value of C2, C5 or C6 and incubated for 72 h. Figure 5 shows that apoptosis (early and late) was the major cellular death modality responsible for the cytotoxicity of DU145 cells upon treatment with dihydroquinazolins. Moreover, treating the cells with 2X IC_50_ value resulted in a more reduction in the number of viable DU145 cells in Q3 particularly with C6 (Fig. 5).

The percentages of apoptosis and necrosis of cell population under different treatment conditions are presented in Fig. 5 and Figure S2 in supplementary. Treating cells with C2, C5 and C6 significantly increased the cells undergoing necrosis and late apoptosis compared to the untreated control at both concentrations (IC_50_ or 2X IC_50_). Approximately 30%, 38% and 41% of the DU145 cells underwent apoptosis (early and late), while 13%, 16% and 15% underwent necrosis when treated with the IC_50_ of C2, C5 and C6, respectively.

### Metabolomics

#### Significantly altered metabolites upon treating DU145 cells with sub-inhibitory concentrations of C5 and C6

The altered metabolites and biochemical pathways upon treating DU145 cells with dihydroquinazolin derivatives was investigated using global metabolomics approach. Two compounds were selected (C5 and C6) including the compound that showed the most potent antiproliferative effect. Representative total ion chromatograms (TIC) of DU145 cells treated with C5, C6 or untreated control are presented in Fig. S3 in supplementary.

##### Multivariate analysis

**Figure 6:**
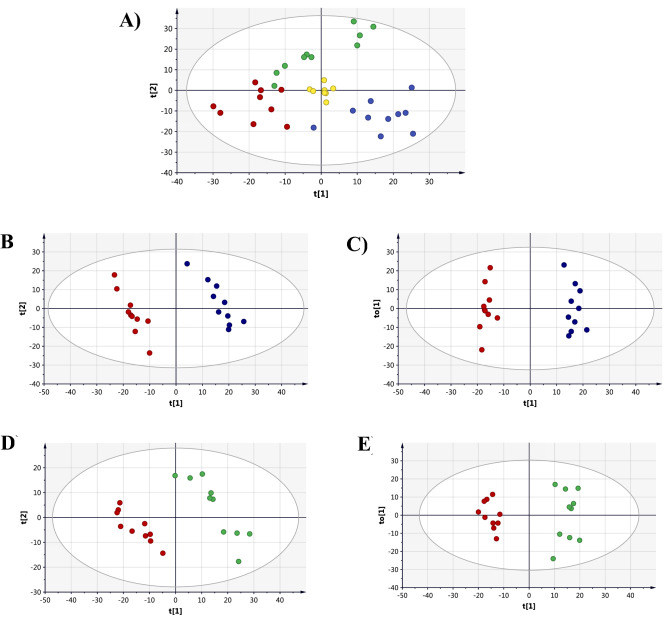
Scores plots of the extracellular metabolic profile of DU145 samples treated with sub-cytotoxic concentrations of C5 (Blue) and C6 (Green). Untreated control samples are in (Red) and QC samples are in (Yellow). (**A**) PCA (R_2_X = 0.637, Q^2^ = 0.28), (**B**) PLS-DA (R2X = 0.36, R2Y = 0.99, Q2 = 0.89) and (**C**) OPLS-DA (R2X = 0.29, R2Y = 0.98, Q2 = 0.86) for C5-treated DU145 cells. (**D**) PLS-DA (R2X = 0.36, R2Y = 0.99, Q2 = 0.82) and (**E**) OPLS-DA (R2X = 0.39, R2Y = 0.95, Q2 = 0.82) for C6-treated DU145 cells.

A total of 1628 mass feature ions were detected using LC–MS/MS analysis, of which 106 metabolites were identified. The unsupervised PCA model revealed a good clustering of the QC samples in the center of the PCA scores plot reflecting satisfactory stability and a valid analytical performance of the LC–MS/MS run (Fig. 6A). Moreover, partial separation and clustering of the three sample groups was evident in the PCA scores plot pointing to the disturbing effect of the two treatments on the extracellular metabolites of the DU145 compared to untreated control group.

To identify significantly altered metabolites upon treating DU145 cells with C5 or C6, supervised analysis using PLS-DA and OPLS-DA was performed (Fig. 6B–E). Each treatment group displayed a clear clustering and separation from the control group. OPLS-DA scores plots had satisfactory R2Y and Q2 values, and passed the permutation validation test (Fig. S4 in supplementary) reflecting robust models. Therefore, OPLS-DA scores plots were used to extract significantly altered features responsible for the class separation noticed. A total of 439 feature ions (of which 49 metabolites were identified), and 313 mass ions (of which 35 metabolites were identified) were significantly altered (VIP > 1) upon treating DU145 with C5 and C6, respectively. The significantly dysregulated metabolites are presented in Tables 1 and 2 in supplementary.

##### Univariate analysis

**Figure 7:**
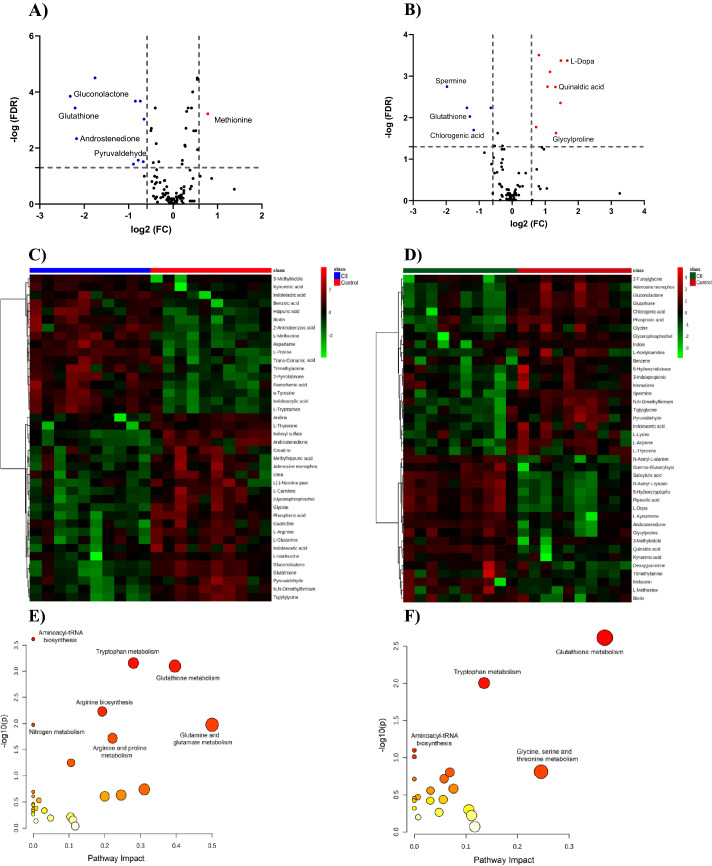
Significantly altered metabolites upon treating DU145 cells with C5 and C6. (**A**) and (**B**) Volcano plots of up (red) and down (blue) regulated metabolites in C5- and C6-treated DU145 cells, respectively, using FDR < 0.05 and FC of 1.5 (**C**) and (**D**) Heatmaps of the level of the top 40 altered metabolites in C5- and C6-treated DU145 cells, respectively. (**E**) and (**F**) Summary of the most affected pathways in C5- and C6-treated DU145 cells, respectively. The node color and size are based on the *P-*value and the pathway impact value, respectively.

In univariate analysis, corrected *P-*value FDR < 0.05 was applied to identify significantly perturbed metabolites based on the binary comparison between each treatment group and controls. The level of 278 mass ions of which 29 ions were identified was significantly altered upon treating DU145 cells with C5. Volcano plot using FDR < 0.05 and FC cutoff of 1.5, revealed that 10 and one metabolites were down- and up-regulated, respectively (Fig. 7A). Heatmap visiluization for the top altered metabolites revealed significant perturbations in the level of several essential and non-essential amino acid including tryptophan, arginine, proline, glutamine, methionine, and tyrosine. Methionine was among the increased metabolites while glycerophosphocholine, carnitine and pyruvaldehyde were decreased in C5-treated DU145 compared to control (Fig. 7C).

Exposing DU145 cells to sub-inhibitory concentration of C6 resulted in the perturbation of the level of 239 feature ions including 17 identified metabolites (Table S2). Volcano plot showed that the level of nine and five metabolites was significantly up- and down-regulated, respectively when using the same previous cut-offs (Fig. 7B). Heatmap visualization of the top 40 altered metabolites in C5-treated DU145 is presented in Fig. 7D. Among the significantly increased metabolites are quinaldic acid and kynurenic acid, while the level of some amino acids (i.e. glutathione, glycine) and spermine was significantly decreased upon treating DU145 cells with C6 (Fig. 7D).

#### Potential biomarkers and altered pathways

The level of 24 and 17 metabolites was significantly altered in both multivariate (VIP > 1) and univariate (FDR < 0.05) analyses in C5 and C6-treated cells, respectively (Tables 1 and 2 in supplementary).

These metabolites highlighted the biochemical pathways perturbed in C5- and C6-treated DU145 cells. Treating DU145 cells with C5 significantly dysregulated aminoacyl-tRNA biosynthesis, tryptophan and glutathione metabolisms, arginine biosynthesis, glutamine and glutamate metabolism and proline metabolism (Fig. 7E). On the other hand, C6 affected a lesser number of pathways, compared to C5, and resulted mainly in disturbances in glutathione metabolism and to a lower extent tryptophan metabolism (Fig. 7F).

## Discussion

Quinazoline derivatives are promising pharmacological compounds and are the building blocks of many pharmaceutical products. They possess a broad spectrum of pharmacological activities with minimum side effects^7,8^. In this work, the effect of synthesized 2,3-dihydroquinazolin-4(1*H*)-one analogues on PC cell lines (DU145 and PC-3) proliferation, migration, adhesion and invasion was investigated for the first time. Additionally, MS based metabolomics profiling was used to identify the molecular mechanisms and the altered biochemical pathways upon treating DU145 cancer cells with two 2,3-dihydroquinazolin derivatives; C5 and C6.

In vitro and in vivo studies have shown that quinazoline derivatives exhibit antitumor activity on PC cell lines^14,29,30^. Herein, the seven compounds showed sufficient cytotoxicity against the two cancer cell lines with C2 and C5 having the most potent effect with IC_50_ < 15 µM against both PC3 and DU145 (Table 1). The significant cytotoxicity of the title compounds C2 and C5 is attributed to the presence of the 3-iodo group and 4-dimethyl amino group on the phenyl ring, which is at the second position of 2,3-dihydroquinazolin-4(1*H*)-one, respectively.

Cancer metastasis is the leading cause of death from cancer^31^. Cell migration, adhesion and invasion are pivotal processes in PC metastasis and malignancy, and are relevant phenotypes when studying the effect of novel therapeutic drugs^32^. Cell migration is the initial phase of cancer cell metastasis, and is crucial in a range of physiological processes including wound healing, tumor invasion and angiogenesis^32^. Individual cells in normal epithelial cells are tightly bound together through numerous junctional organelles. For this reason, epithelial cells often lack the motility and invasiveness of cells of mesenchymal origin. As a result, it is possible that the formation of malignant carcinomas from normal epithelia disrupts the intercellular adhesion system^33^. Our results revealed that compared to control all seven compounds significantly suppressed DU145 and PC3 migration after 48 and 72 h. Moreover, both C2 and C5 successfully inhibited PC cell adhesion and invasion.

The data presented herein highlight the importance of these compounds as potential anticancer agents particularly C5 which had the most potent cytotoxic effect, exhibited the highest inhibition of wound closure and showed promising anti-adhesive and anti-invasive effects. It is worth mentioning that the safety of these compounds was evaluated against normal fibroblast cells and all compounds showed significantly higher cytotoxicity against PC cell lines compared to fibroblast^34^. The most promising compound, C5, exhibited 15 and 28 times higher IC_50_ in fibroblast compared to DU145 and PC3, respectively.

Cell cycle analysis was performed to explore the influence of three representative compounds (C2, C5 and C6) on the cell cycle progression of DU145 prostate cell, and to determine their apoptosis/necrosis status. The findings revealed that apoptosis (early and late) was the major cellular death modality responsible for decreasing DU145 cells proliferation. Our results are in line with previous reports where apoptosis was the predominant form of cell death caused by chemotherapeutic agents in PC^35–37^. Targeting apoptosis has been proposed as a viable and promising approach for the development of novel cancer therapies^35–37^.

Cancer cells have altered metabolism, which has been extensively proven. Variations in metabolite contents in response to cell treatment indicate changes in metabolic enzyme activity in cancer cells, and hence will reflect the underlying pathways affected by cell treatment and might point to potential therapeutics target. Metabolomics is a comprehensive and reliable analytical method that identifies the sequence of overall biological changes in the cellular metabolism caused by internal and external factors. In the presented work, an LC/MS-based metabolomics approach was used to explore the pathways and biochemical mechanisms altered upon treating DU145 cells with sub-inhibitory concentrations of two dihydroquinazolin derivative; C5 and C6, which in turn might be linked to their cytotoxic effect.

**Figure 8:**
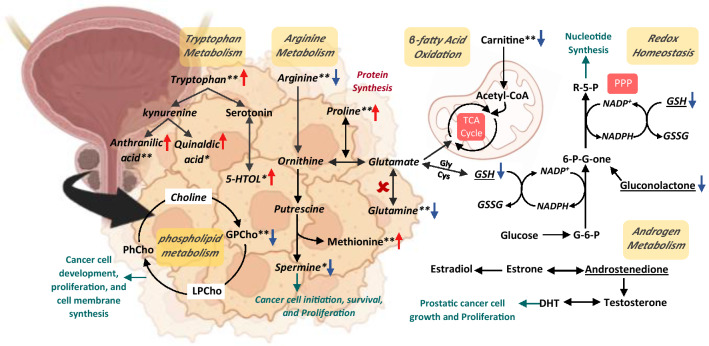
A scheme representation of the different intermediates affected pathways in DU145 cells after treatment with C5 and C6. PPP: pentose phosphate pathway, GSH: glutathione, GSSG: oxidized glutathione, DHT: dihydrotestosterone, G-6-P: Glucose 6-phosphate, 6-P-G-one: 6-phosphogluconate, r-5-P, SAM: S-Adenosyl methionine, 5-HTOL: 5-hydroxytryptophan, PhCho: phosphatidylcholine, LPCho: lysophosphatidylcholine, and GPCho: Glycerophosphocholine. Red arrows indicate upregulation, while blue arrows indicate down regulation.^**^Significant in DU145 cells treated with C5 only, ^*^Significant in DU145 cells treated with C6 only, and underlined metabolite is significant in both C5 and C6.

Both C5 and C6 showed significant dysregulation in several metabolites involved in tryptophan metabolisms such as tryptophan and 2-aminobenzoic acid (anthranilate) in C5-treated cells, and quinaldic acid and 5-hydroxytryptophan in C6-treated cells (Table S1 and S2), Fig. 8. Tryptophan metabolism is an essential metabolic pathway for the augmentation of tumor intrinsic malignancy and limiting anti-tumor immunity and targeting tryptophan metabolism and kynurenine pathway has been considered an effective cancer immunotherapy to retard cancer growth^38^.

Moreover, treating DU145 cells with either C5 or C6 has demonstrated a disruption in the redox homeostasis process evident by the significant reduction in the pentose phosphate pathway (PPP) intermediate metabolite gluconolactone, and the tripeptide glutathione (GSH), Fig. 8. Several essential pathways are utilized in cancer cells to maintain redox homeostasis and generate NADPH, including the PPP^39^ and the antioxidant glutathione system^40^. PPP is a branch of glycolysis, that encompasses several oxidation and hydrolysis steps starting with the oxidation of glucose-6-phosphate and ending with the generation of ribulose-5-phosphate in concomitant reduction of NADP + to NADPH^39,41^. NADPH is a key element in maintaining GSH in its reduced form and thus opposing oxidative stress which promotes cancer growth^42^. Herein, hindering the redox balance inside DU145 cells might retard cell proliferation leading to cancer cell death. A previous study has reported cell cycle arrest, tumor regression and death in prostate carcinoma allografts as well as xenografts after depleting these cells from GSH by the action of a cyst(e)inase enzyme administration^43^.

Besides altering redox hemostasis, treating DU145 with C6 significantly reduced the level of the polyamine and spermine, compared to the untreated cells. Spermine is produced from the metabolism of ornithine, one of the urea cycle intermediates, Fig. 8, and has been correlated with different cancers diagnosis, progression and initiation^44^. Several studies demonstrated that depleting the polyamines pool in prostatic cells and introducing polyamines inhibitors have induced cell arrest and apoptosis^45–48^. Our findings support the previously reported promising role of spermine and other polyamines as key biomarkers and potential target for PC chemotherapy^49,50^.

Exposure of DU145 to C5 induced perturbations in a larger number of biochemical processes including methionine, glutamine, proline and arginine metabolisms. Methionine plays a crucial role in polyamine and glutathione synthesis, and DNA methylation which are crucial for tumor proliferation and progression^51^. The other amino acids are crucial nitrogen sources for tumor growth and are involved in multiple metabolic pathways^52^. In cancer, a second Warburg-like effect to glucose metabolism reprogramming is represented by the significant remodeling of glutamine nitrogen metabolism to synthesize different nucleotides and amino acids to support the high energy requirements and proliferation demands^53^. Arginine also regulates different aspects of tumorigenesis such as inflammation, cell growth and the synthesis of proline, polyamines and proteins^54,55^. Glutamine metabolism is highly upregulated by different oncogenic signals and is associated with the progression, survival and growth of PC^56–58^, while arginine is overexpressed in many tumors including PC^59^. Herein, C5 significantly decreased the level of both amino acids which might contribute to its potent cytotoxic effect in DU145 cells.

Fatty acid oxidation (β-FAO) is a dominant pathway for bioenergetic supply for PC proliferation and growth^60^. An important step in β-FAO is the transportation of long-chain fatty acids by carnitine for subsequent β- oxidation and energy production^61,62^. C5 treatment significantly reduced the level of carnitine and thus deprived the DU145 cells of a crucial source of energy leading to decreased cell proliferation.

In addition, C5 intervention led to a reduction in glycerophosphocholine level, an essential metabolite in the choline phospholipid metabolism cycle, Fig. 8. Activated choline metabolism has been a hallmark in most cancers including PC. Compared to normal prostatic cells, PC cells have increased levels of phosphocholine, glycerophosphocholine, and total choline-containing compounds^63–66^. Cancer cells require a massive amount of choline containing to serve as substrates for membrane growth during cell proliferation^67,68^. The upregulation of the choline metabolism has been strongly linked to tumor progression and metabolic reprogramming in cancer^69,70^. Therefore, inhibition of this cycle has been reported to have an antiproliferative effect on different cancer cell lines^71^.

It is worth mentioning that while DU-145 cells are considered androgen-independent cells^72^, a perturbation in the androgen metabolism was detected upon treating the cells with C5 and C6, Fig. 8. Although the majority of human prostate cancer cell lines are said to be AR-negative, multiple studies have found that the AR mRNA is expressed at measurable quantities in the PC-3 and DU-145 prostate cancer cell lines. Furthermore, the expression of AR protein in both DU-145 and PC-3 cell lines has been shown utilizing whole cell binding tests to androgen ligand^73^. Androgens are necessary hormones for the growth and the maintenance of the male reproductive system. Despite being a weak androgen steroid, androstenedione (AD) is an essential step in the biosynthesis of both testosterone and estrogen^74^. PC cells tend to convert AD to dihydrotestosterone (DHT); a potent ligand for the activation of androgen receptors (ARs)^75^. The latter are the lead cellular metabolism regulators in PC that fuel its proliferation and progression. Moreover, ARs control several biochemical processes including glycolysis, fatty acids metabolism, mitochondrial respiration and amino acids, polyamines and nucleotides synthesis^76–79^. Herein, C5 and C6 treatment have shown accumulation of AD which might reflect a dysfunction in the enzyme responsible for its conversion to DHT and hence might affect the activity of AR leading to metabolic changes in PC cells. The effect of the compounds on AR-positive cell lines remains urgent to confirm this finding.

## Conclusion

Quinazoline derivatives are promising pharmacological compounds with minimum side effects. In the present study, the effect of seven synthesized 2,3-dihydroquinazolin-4(1*H*)-one analogues as potential anticancer agent against PC cell lines (DU145 and PC-3) was investigated for the first time. The compounds demonstrated sufficient antiproliferative and promising antimigration effects against the two PC cell lines. C5 displayed the most potent cytotoxic effect with IC_50_ < 5 µM against both cell lines, and successfully inhibited PC cell adhesion and invasion. Metabolomics study revealed that treating DU145 cells with subtoxic concentration of C5 altered several biochemical processes essential for cancer cell proliferation, progression and growth including redox homeostasis, amino acids metabolisms, energy production and choline phospholipid metabolism.

The data herein highlighted the importance of dihydroquinazolin-4(1*H*)-one analogues as potential anticancer agents particularly C5, and supported the important role of LC–MS metabolomics in identifying new therapeutic targets and mechanisms by which compounds can exert their potential cytotoxic effect. The effect of the seven compounds on androgen-dependent cells (androgen positive cell lines) and against normal primary prostate epithelial cells are to be investigated in a future study. Besides, targeted MS analysis will be performed to validate the effect of C5 on specific pathways including glutamine and fatty acid oxidation and to evaluate the underlying biochemical pathways disturbed in dihydroquinazolinone analogues treated-PC3 cell lines. In vivo study using animal model is warranted to evaluate the effectiveness of the compounds as potential anticancer agent and to reflect the long-term effect.

## Supplementary Information

Supplementary Figures.

Supplementary Tables.

## Data Availability

The datasets used and/or analysed during the current metabolomics study available from the corresponding author on reasonable request. All other data generated or analysed during this study are included in this published article [and its supplementary information files].

## References

[1] Rawla P (2019). Epidemiology of prostate cancer. World J. Oncol..

[2] Bray F (2018). Global cancer statistics 2018: GLOBOCAN estimates of incidence and mortality worldwide for 36 cancers in 185 countries. Ca-a Cancer J. Clin..

[3] Crawford ED (2003). Epidemiology of prostate cancer. Urology.

[4] Litwin MS, Tan HJ (2017). The diagnosis and treatment of prostate cancer a review. Jama-J. Am. Med. Assoc..

[5] De Silva F, Alcorn J (2022). A tale of two cancers: A current concise overview of breast and prostate cancer. Cancers.

[6] Eckford PDW, Sharom FJ (2009). abc efflux pump-based resistance to chemotherapy drugs. Chem. Rev..

[7] Karan R (2021). Recent advances on quinazoline derivatives: A potential bioactive scaffold in medicinal chemistry. Chemengineering.

[8] Wang D, Gao F (2013). Quinazoline derivatives: Synthesis and bioactivities. Chem. Cent. J..

[9] Asif, M., Chemical characteristics, synthetic methods, and biological potential of quinazoline and quinazolinone derivatives*.* Int. J. Med. Chem. **2014 **(2014). 10.1155/2014/395637 PMC4321853 25692041

[10] Shagufta, Ahmad I., An insight into the therapeutic potential of quinazoline derivatives as anticancer agents*.* Medchemcomm **8**(5): 871–885 (2017). 10.1039/c7md00097a PMC6072504 30108803

[11] Venugopala KN (2020). Larvicidal activities of 2-Aryl-2,3-dihydroquinazolin -4-ones against malaria vector anopheles arabiensis, in silico admet prediction and molecular target investigation. Molecules.

[12] Venugopala KN (2022). Antitubercular, cytotoxicity, and computational target validation of dihydroquinazolinone derivatives. Antibiotics.

[13] Gatadi S (2020). Synthesis and evaluation of new 4 (3H)-Quinazolinone derivatives as potential anticancer agents. J. Mol. Struct..

[14] Hour M-J (2012). Antitumor effects of the novel quinazolinone MJ-33: Inhibition of metastasis through the MAPK, AKT, NF-κB and AP-1 signaling pathways in DU145 human prostate cancer cells. Int. J. Oncol..

[15] Jafari E (2016). Quinazolinone and quinazoline derivatives: Recent structures with potent antimicrobial and cytotoxic activities. Res. Pharm. Sci..

[16] Locasale JW, Cantley LC (2010). Altered metabolism in cancer. BMC Biol..

[17] Zhou B (2012). LC-MS-based metabolomics. Mol. BioSyst..

[18] Liu X, Locasale JW (2017). Metabolomics: a primer. Trends Biochem. Sci..

[19] Aleidi S (2021). Obesity connected metabolic changes in type 2 diabetic patients treated with metformin. Front. Pharmacol..

[20] Dahabiyeh LA (2021). Phospholipid-gold nanorods induce energy crisis in MCF-7 cells: Cytotoxicity evaluation using lc-ms-based metabolomics approach. Biomolecules.

[21] Dahabiyeh LA (2021). A metabolic pattern in healthy subjects given a single dose of metformin: A metabolomics approach. Front Pharmacol.

[22] Johnson CH, Ivanisevic J, Siuzdak G (2016). Metabolomics: Beyond biomarkers and towards mechanisms. Nat. Rev. Mol. Cell Biol..

[23] Dudka I (2020). Comprehensive metabolomics analysis of prostate cancer tissue in relation to tumor aggressiveness and TMPRSS2-ERG fusion status. BMC Cancer.

[24] Frahm AB (2020). Stable isotope resolved metabolomics classification of prostate cancer cells using hyperpolarized NMR data. J. Magn. Reson..

[25] Kdadra M (2019). Metabolomics biomarkers of prostate cancer: A systematic review. Diagnostics.

[26] Yin PY (2009). A metabonomic study of hepatitis B-induced liver cirrhosis and hepatocellular carcinoma by using RP-LC and HILIC coupled with mass spectrometry. Mol. BioSyst..

[27] Pang Z (2021). MetaboAnalyst 5.0: Narrowing the gap between raw spectra and functional insights. Nucleic Acids Res..

[28] Xia J, Wishart DS (2011). Web-based inference of biological patterns, functions and pathways from metabolomic data using metaboanalyst. Nat. Protoc..

[29] Batty M (2016). The role of alpha 1-adrenoceptor antagonists in the treatment of prostate and other cancers. Int. J. Molecul. Sci..

[30] Bilbro J, Mart M, Kyprianou N (2013). Therapeutic value of quinazoline-based compounds in prostate cancer. Anticancer Res..

[31] Novikov NM (2021). Mutational drivers of cancer cell migration and invasion. Br. J. Cancer.

[32] Pijuan, J., et al., In vitro cell migration, invasion, and adhesion assays: From cell imaging to data analysis. Front. Cell Dev. Biol. **7 **(2019). 10.3389/fcell.2019.00107 PMC6587234 31259172

[33] Behrens J (1993). The role of cell adhesion molecules in cancer invasion and metastasis. Breast Cancer Res. Treat..

[34] Venugopala KN (2022). Antitubercular, cytotoxicity, and computational target validation of dihydroquinazolinone derivatives. Antibiotics.

[35] Shapiro GI, Harper JW (1999). Anticancer drug targets: Cell cycle and checkpoint control. J. Clin. Investig..

[36] Pfeffer CM, Singh ATK (2018). Apoptosis: A target for anticancer therapy. Int. J. Molecul. Sci..

[37] McKenzie S, Kyprianou N (2006). Apoptosis evasion: The role of survival pathways in prostate cancer progression and therapeutic resistance. J. Cell. Biochem..

[38] Peyraud, F., et al., Targeting tryptophan catabolism in cancer immunotherapy era: Challenges and perspectives. Front. Immunol. **13 **(2022). 10.3389/fimmu.2022.807271 PMC8841724 35173722

[39] Batsios, G., et al., Imaging 6-phosphogluconolactonase activity in brain tumors in vivo using hyperpolarized δ-[1–13C]gluconolactone. Front. Oncol. **11 **(2021). 10.3389/fonc.2021.589570 PMC8082394 33937017

[40] Li P (2016). Redox homeostasis protects mitochondria through accelerating ROS conversion to enhance hypoxia resistance in cancer cells. Sci. Rep..

[41] Jiang P, Du W, Wu M (2014). Regulation of the pentose phosphate pathway in cancer. Protein Cell.

[42] Chen L (2019). NADPH production by the oxidative pentose-phosphate pathway supports folate metabolism. Nat. Metab..

[43] Cramer SL (2017). Systemic depletion of L-cyst(e)ine with cyst(e)inase increases reactive oxygen species and suppresses tumor growth. Nat. Med..

[44] Tse RT-H (2022). The potential role of spermine and its acetylated derivative in human malignancies. Int. J. Mol. Sci..

[45] Pegg AE, Lockwood DH, Williams-Ashman HG (1970). Concentrations of putrescine and polyamines and their enzymic synthesis during androgen-induced prostatic growth. Biochem. J..

[46] Eiseman JL (1998). Tumor-targeted apoptosis by a novel spermine analogue, 1,12-diaziridinyl-4,9-diazadodecane, results in therapeutic efficacy and enhanced radiosensitivity of human prostate cancer1. Can. Res..

[47] Schipper RG, Penning LC, Verhofstad AAJ (2000). Involvement of polyamines in apoptosis. Facts and controversies: Effectors or protectors?. Semin. Cancer Biol..

[48] Meyskens FL, Simoneau AR, Gerner EW (2014). Chemoprevention of prostate cancer with the polyamine synthesis inhibitor difluoromethylornithine. Recent Results Cancer Res..

[49] Schipper RG (2003). Polyamines and prostatic cancer. Biochem. Soc. Trans..

[50] Peng Q (2021). The emerging clinical role of spermine in prostate cancer. Int. J. Mol. Sci..

[51] Wanders D, Hobson K, Ji X (2020). Methionine restriction and cancer biology. Nutrients.

[52] Kurmi K, Haigis MC (2020). Nitrogen metabolism in cancer and immunity. Trends Cell Biol..

[53] Kodama M, Nakayama KI (2020). A second warburg-like effect in cancer metabolism: The metabolic shift of glutamine-derived nitrogen. BioEssays.

[54] Matos A (2021). Arginine and arginases modulate metabolism, tumor microenvironment and prostate cancer progression. Nutrients.

[55] Wang B (2018). Phospholipid remodeling and cholesterol availability regulate intestinal stemness and tumorigenesis. Cell Stem Cell.

[56] Xu L (2022). Targeting glutamine metabolism network for the treatment of therapy-resistant prostate cancer. Oncogene.

[57] White MA (2017). Glutamine transporters are targets of multiple oncogenic signaling pathways in prostate cancer. Mol. Cancer Res..

[58] He Z (2022). HepaCAM-PIK3CA axis regulates the reprogramming of glutamine metabolism to inhibit prostate cancer cell proliferation. Int. J. Oncol..

[59] Mumenthaler SM (2008). Expression of arginase II in prostate cancer. Int. J. Oncol..

[60] Liu Y (2006). Fatty acid oxidation is a dominant bioenergetic pathway in prostate cancer. Prostate Cancer Prostatic Dis..

[61] Jin L, Alesi GN, Kang S (2016). Glutaminolysis as a target for cancer therapy. Oncogene.

[62] Melone MAB (2018). The carnitine system and cancer metabolic plasticity. Cell Death Dis..

[63] Hanahan D, Weinberg RA (2011). Hallmarks of cancer: The next generation. Cell.

[64] Ackerstaff E (2001). Detection of increased choline compounds with proton nuclear magnetic resonance spectroscopy subsequent to malignant transformation of human prostatic epithelial cells. Cancer Res..

[65] Griffiths JR (1981). 31P-NMR investigation of solid tumours in the living rat. Biosci. Rep..

[66] Daly PF (1987). Phospholipid metabolism in cancer cells monitored by 31P NMR spectroscopy. J. Biol. Chem..

[67] Fagone P, Jackowski S (2013). Phosphatidylcholine and the CDP-choline cycle. Biochim. Biophys. Acta.

[68] Ridgway ND (2013). The role of phosphatidylcholine and choline metabolites to cell proliferation and survival. Crit. Rev. Biochem. Mol. Biol..

[69] Moestue S (2011). HR MAS MR spectroscopy in metabolic characterization of cancer. Curr. Top. Med. Chem..

[70] Haukaas TH (2017). Metabolic portraits of breast cancer by HR MAS MR spectroscopy of intact tissue samples. Metabolites.

[71] Lacal JC, Campos JM (2015). Preclinical characterization of RSM-932A, a novel anticancer drug targeting the human choline kinase alpha, an enzyme involved in increased lipid metabolism of cancer cells. Mol. Cancer Ther..

[72] Bluemn EG (2017). androgen receptor pathway-independent prostate cancer is sustained through FGF signaling. Cancer Cell.

[73] Alimirah F (2006). DU-145 and PC-3 human prostate cancer cell lines express androgen receptor: Implications for the androgen receptor functions and regulation. FEBS Lett..

[74] Badawy MT (2021). Androstenedione (a natural steroid and a drug supplement): A comprehensive review of its consumption, metabolism, health effects, and toxicity with sex differences. Molecules.

[75] Auchus RJ, Sharifi N (2020). Sex hormones and prostate cancer. Annu. Rev. Med..

[76] Massie CE (2011). The androgen receptor fuels prostate cancer by regulating central metabolism and biosynthesis. Embo. J..

[77] Bader DA, McGuire SE (2020). Tumour metabolism and its unique properties in prostate adenocarcinoma. Nat. Rev. Urol..

[78] Awad D (2018). Delineation of the androgen-regulated signaling pathways in prostate cancer facilitates the development of novel therapeutic approaches. Curr. Opin. Pharmacol..

[79] Uo, T., Sprenger, C.C., Plymate, S.R. Androgen receptor signaling and metabolic and cellular plasticity during progression to castration resistant prostate cancer*.* Front. Oncol., **10 **(2020). 10.3389/fonc.2020.580617 PMC7581990 33163409

