# Supplementary material for: Molecular and metabolic alterations of 2,3-dihydroquinazolin-4(1H)-one derivatives in prostate cancer cell lines
Source: Sci Rep. 2022 Dec 14;12:21599. doi: 10.1038/s41598-022-26148-4 (PMC9751122; doi:10.1038/s41598-022-26148-4)
Supplement: Supplementary file 1 — Supplementary Figures. [file 41598_2022_26148_MOESM1_ESM.docx]

**Molecular and Metabolic Alterations of 2,3-Dihydroquinazolin-4(1*H*)-one Derivatives in Prostate Cancer Cell Lines**

Lina A. Dahabiyeh^1*^, Wafa Hourani^2^, Wesam Darwish^1^, Farah Hudaib^3^, Bashaer Abu-Irmaileh^4^, Pran Kishore Deb^2^, Katharigatta N. Venugopala^5,6^, Viresh Mohanlall^6^, Rana Abu-Dahab^7^, Mohammad H. Semreen^8^​, Yasser Bustanji^9^

^1^Department of Pharmaceutical Sciences, School of Pharmacy, The University of Jordan, Amman, 11942, Jordan

^2^Department of Pharmaceutical Sciences, Faculty of Pharmacy, Philadelphia University, Amman 19392, Jordan

^3^Depatment of Pharmaceutical Chemistry, School of Pharmaceutical Sciences, the Hashemite University, Zarqa, 13133, Jordan

^4^Hamdi Mango Center for Scientific Research, The University of Jordan, 11942 Amman, Jordan

^5^Department of Pharmaceutical Sciences, College of Clinical Pharmacy, King Faisal University, Al-Ahsa 31982, Saudi Arabia

^6^Department of Biotechnology and Food Science, Faculty of Applied Sciences, Durban University of Technology, Durban 4000, South Africa

^7^Department of Biopharmaceutics and Clinical Pharmacy, School of Pharmacy, The University of Jordan, Amman 11942, Jordan

^8^Department of Medicinal Chemistry, College of Pharmacy, University of Sharjah, Sharjah 27272, UAE

^9^Department of Basic Medical Sciences, College of Medicine, University of Sharjah, Sharjah 27272, UAE

***Correspondence**

Lina A. Dahabiyeh

Department of Pharmaceutical Sciences, School of Pharmacy, The University of Jordan

Queen Rania St, Amman 11942, Jordan.

Telephone: 00962 6 5355000 ext. 23304

Email: l.dahabiyeh@ju.edu.jo

**List of Supplementary Figures**

|  | |  |  |
| --- | --- | --- | --- |
| **Figure S1:** Dose-response curves of the seven derivatives against PC3 (black) and DU450 (Red) cell lines. A, B, C, D, E, F, and G refer to compounds 1, 2, 3, 4, 5, 6, and 7 as indicated in Table 1. Each concentration point represents the average of three experimental replicates. | |  |  |
|  | |  |  |
|  | |  | |
|  | | | |
|  | |  | |
|  | |  | |
| **Figure S2.** Dihydroquinazolins (C2, C5, and C6) induced apoptosis and necrosis in DU145 cells. Data presented as mean% of cells ± SEM in each group of necrotic cells (A), cells in late apoptosis (B), and cells in early apoptosis (C). ANOVA test followed by Tukey’s test was performed. ^*^ *P*-value ≤ 0.05, ^**^ *P*-value ≤ 0.01, ^***^ *P*-value ≤ 0.001. | | | |

| **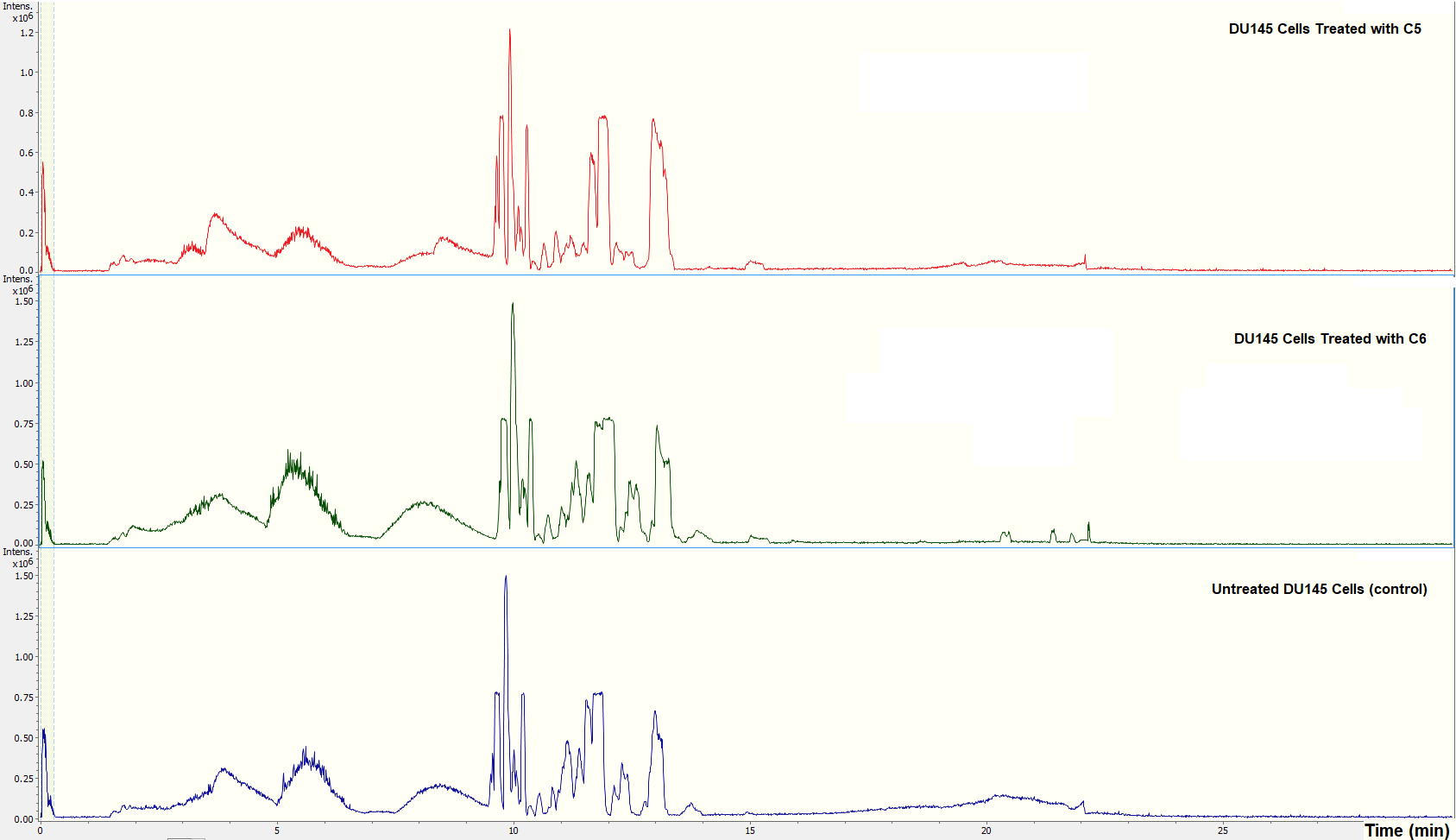** |
| --- |
| **Figure S3.** Representative total ion chromatograms (TIC) of LC-MS/MS metabolomics study. TIC from DU145 cells treated with C5, C6 and untreated controls are presented in red, green and blue respectively. |

|  |
| --- |
| 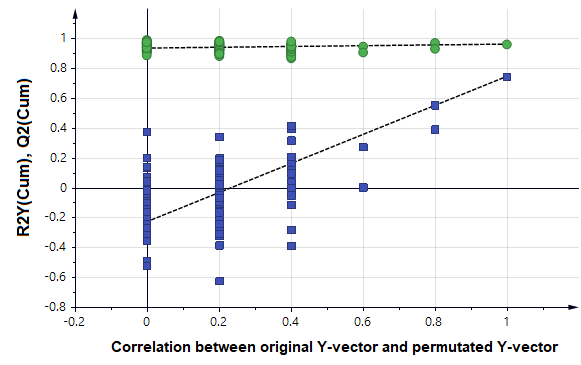 |
|  |
| 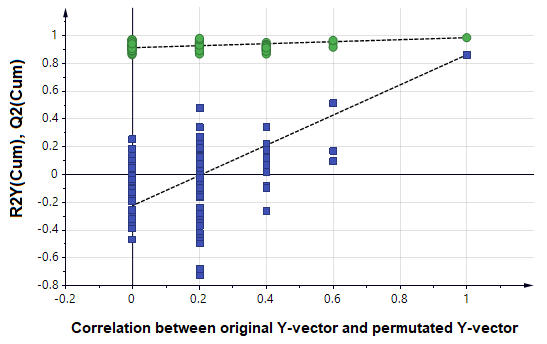 |
| **Figure S4.** Permutation test for the validation of the OPLS-DA model generated from the binary comparison between (A) C5 treated DU145 group and control (untreated) group, and (B) C6 treated DU145 group and control (untreated) group. Q^2^ (blue squares) and R^2^Y (green circles). Values from the permuted analysis models (left- side) should be less than the initial generated model values (right- corner). |
